# Knowledge of, and attitudes to giving expressed breastmilk to infants in rural coastal Kenya; focus group discussions of first time mothers and their advisers

**DOI:** 10.1186/s13006-018-0158-9

**Published:** 2018-04-30

**Authors:** Alison W. Talbert, Benjamin Tsofa, Edward Mumbo, James A. Berkley, Martha Mwangome

**Affiliations:** 1 Centre for Geographic Medicine (Coast), Kenya Medical Research Institute, P.O. Box 230, Kilifi, 80108 Kenya; 2 Kwale County Department of Health, P.O Box 4, Kwale, 80403 Kenya; 3 Centre for Tropical Medicine and Global Health, Nuffield Department of Medicine Research Building, University of Oxford, Old Road Campus, Roosevelt Drive, Oxford, OX3 7FZ UK

**Keywords:** Mothers, Expressed breastmilk, Milk supply, Knowledge, Safety, Grandmothers

## Abstract

**Background:**

The World Health Organization (WHO)/UNICEF Baby-Friendly Hospital Initiative step number five of the “Ten steps to successful breastfeeding” states “Show mothers how to breastfeed and how to maintain lactation even if they should be separated from their infants.” Urban mothers in Nairobi have low rates of exclusive breastfeeding after returning to work but there are no published data on rural Kenya mothers’ infant feeding practices when working or schooling away from home.

**Methods:**

We explored knowledge of, and attitudes to, the practice of giving expressed breastmilk in a mixed methods observational study of breastfeeding in rural Kenyan mothers. Fifty mothers with newborns, identified by nurses and community health workers, were asked questions about their experiences of breastfeeding and who they had sought or received advice from on breastfeeding. Focus group discussions, one with community health workers, and four each with mothers and their named advisers were held. Recordings were analyzed using a thematic framework approach.

**Results:**

The main themes were: the baby’s right to feed from the breast, lack of knowledge about expressing and giving breastmilk, negative attitudes towards expressed breastmilk, and traditional customs for disposing of expressed breast milk. Most participants did not have any experience of giving expressed breastmilk to infants. They described practices of expressing and discarding milk when the mother or baby was ill, to relieve discomfort from engorgement or after the baby had died.

**Conclusions:**

Feeding expressed breastmilk to infants is a new concept in this context. Promotion of, and training in this practice would help mothers to maintain their milk supply when away from their babies and benefit the infants of working and schoolgirl mothers.

## Background

The WHO Global Strategy for Infant and Young Child Feeding recommends planning and monitoring of the Baby-Friendly Hospital Initiative and expanding it beyond the maternity care setting [1]. Step number five of the “Ten steps to successful breastfeeding” is “Show mothers how to breastfeed and how to maintain lactation even if they should be separated from their infants.”

Kenya has high breastfeeding rates, with 99% of babies ever breastfed but only 61% are exclusively fed for the first 6 months of life [2]. By the age of four to 5 months 15% of infants are given other milks in addition to breastmilk and 27% have started receiving complementary foods [2]. Formula milk is expensive compared to most household incomes in Kenya so that animal milk from locally kept livestock, e.g. cows, goats or camels, is more likely to be given. Cows’ milk given in early infancy can lead to iron-deficiency anaemia secondary to gastro-intestinal tract irritation and blood loss, and dehydration secondary to high renal solute load [3]. Mixed fed infants under 6 months have a higher risk of diarrhoeal diseases as well as malnutrition [4, 5].

Mothers’ milk is the ideal food to meet infants’ nutritional requirements in the first 6 months of life but separation of the mother from her baby for periods of more than a few hours requires her to be able to express her milk and store it hygienically for caretakers to give. The skill of breast milk expression needs to be acquired early before return to work or education, and caretakers must be prepared to handle and give mothers’ milk in preference to other milks and baby foods in the first 6 months.

A recent study of urban mothers living in informal settlements in Nairobi has shown low uptake of the WHO recommendations on breastfeeding with very few working mothers expressing breast milk (EBM) for caretakers to give their infants [6]. Poor urban mothers in the informal labour market do not receive paid maternity leave and thus they are forced to return to work leaving their infants, sometimes even in the first month of life, with caretakers or in day care nurseries. These babies are given porridge and liquids other than breastmilk. There is little information available on the infant feeding strategies that rural mothers in Kenya use when leaving their young infants with caretakers in order to work or return to education.

We conducted a mixed methods study of advice on infant feeding given to first-time mothers living in a rural area of Kilifi County, which has a high prevalence of teenage pregnancies (21.8%), on the coast of Kenya [7]. We incorporated questions about expressing and giving expressed breastmilk into focus group discussions with the mothers and their named advisers. The aim was to learn more about their experience of and attitudes towards maintaining milk supply and ensuring exclusive breastfeeding in the first 6 months of life, particularly during times when the mother was separated from her baby for more than a few hours.

## Methods

The main objective of the study was to determine the problems that first-time mothers experienced when breastfeeding and how they solved them. The participants were recruited from the population of approximately 18,000 residents served by two government health facilities in Jaribuni Division, a rural area about 30 km inland of Kilifi town where the nearest hospital is situated. It was estimated that approximately 90 women living in Jaribuni Division would become first-time mothers within a year. The study aimed to recruit 50 first-time mothers for a descriptive study. Community sensitization meetings were held prior to the study. Local administration leaders, dispensary nurses and community health workers (CHWs) were asked to notify the study team of first-time mothers with newborn babies. Identified mothers were first visited by the CHW, who explained the aims of the study and for those mothers who agreed to participate, a date was booked for a second visit. At the second visit, mothers were seen at home by the CHW and the principal investigator. Written informed consent was obtained by the CHW in the mother’s preferred language. A questionnaire was administered by the CHW on demographic characteristics; current infant feeding practices, including breastfeeding initiation and challenges; and key advisers on breastfeeding problems.

Focus group discussions (FGD) were held with study participant mothers and their named advisers who were purposively selected for different groups according to marital status of the mothers and advisers’ relationships to the index mothers. The topics were first tested on a group of local CHWs and included knowledge, attitudes and practices of mothers and their advisers, usually the babies’ grandmothers, around exclusive breastfeeding for the infant’s first 6 months of life. We probed their knowledge of the practice of feeding infants with expressed breast milk and elicited their views on when it could be used. The study methods, questionnaire and focus group discussion topics were fully described in a previous publication [7].

The focus group discussions were moderated by an experienced research assistant from the Kenya Medical Research Institute (KEMRI)-Wellcome Trust Research Programme and recorded for later transcription and translation into English. The principal investigator assisted the transcriber, who had also been present at the focus group discussions as note taker, with translation into English. The data were entered onto Nvivo10 software; two researchers read and independently coded the transcripts, then met to agree on an analytical framework [8]. Categories of related codes were summarized using framework matrices in order to examine the similarities within and between the discussion groups. Overarching themes were sought using a combination of deductive and inductive analysis.

## Results

Nine focus group discussions, starting with a pilot group with community health workers (*n* = 10), and four each with mothers (*n* = 21) and their advisers (*n* = 23), were held in the vernacular language, Kikauma, between November 2014 and February 2015. The community health workers’ group comprised of eight women and two men, with an age range of 27 to 46 years (median age 40 years) and all had attended primary school and one also secondary school. The mothers’ and advisers’ ages, educational status and stated occupation are listed in Tables 1 and 2 respectively. It was noted that most mothers had not completed their primary school education (8 years) and only one had finished secondary school (4 years) but very few advisers had any formal education at all.

**Table 1:** Sociodemographic characteristics of mothers’ focus group discussion participants

Focus group	Age	Education type	Highest level of education (class)	Occupation
M1	15	Primary	7	None
M1	16	Primary	7	None
M1	19	Primary	8	None
M1	16	Primary	6	None
M1	16	Primary	5	None
M1	19	Primary	5	None
M2	18	Primary	7	Housewife
M2	23	Primary	8	Housewife
M2	24	Primary	8	Businesswoman
M2	24	Secondary	4	Housewife
M2	18	Primary	7	Housewife
M3	18	Primary	7	Housewife
M3	20	Primary	8	Businesswoman
M3	17	Primary	7	Housewife
M3	18	Primary	8	Housewife
M3	22	Primary	2	Housewife
M4	22	Primary	8	Housewife
M4	20	Primary	4	Housewife
M4	29	Primary	8	Dressmaking
M4	19	Primary	7	Housewife
M4	19	Primary	7	Housewife

**Table 2:** Sociodemographic characteristics of advisers’ focus group discussion participants

Focus group	Age	Education type	Highest level of education (class)	Occupation
A1	Not known	None	0	Farmer
A1	47	None	0	Farmer
A1	40	None	0	Farmer
A1	48	None	0	Farmer
A1	Not known	Adult education	1	Farmer
A1	Not known	None	0	Farmer
A1	34	Primary	7	Businesswoman
A2	52	None	0	Farmer
A2	Not known	None	0	Farmer
A2	Not known	None	0	Farmer
A2	Not known	None	0	Farmer
A2	Not known	None	0	Farmer
A2	Not known	None	0	Farmer
A2	30	Primary	8	Farmer
A3	Not known	None	0	Farmer
A3	Not known	None	0	Farmer
A3	Not known	None	0	Farmer
A3	55	Primary	7	Businesswoman
A4	Not known	None	0	Charcoal seller
A4	Not known	None	0	Farmer
A4	47	None	0	Farmer
A4	Not known	None	0	Farmer
A4	36	None	0	Farmer

From the focus group discussions, four main themes on expressing breastmilk emerged. Firstly, it was perceived that the baby has a right to feed directly from the breast, secondly was the lack of knowledge and skills to express, store and give breastmilk, thirdly was a negative attitude towards expressed breastmilk, and lastly were traditional customs around expressing breast milk (when and how it should be done).

### The baby’s right to feed from the breast

The advisers who were mostly grandmothers held strong views on the baby’s right to suckle at the breast and considered it vital for proper child development:

*Participant 2: How will you give him (expressed milk) and you want to teach him how to eat?*

*Participant 4: He must suck to open his jaws*

*Participant 4: Yes jaws, if he is unable to suck then you express but if he can suck you leave him to suck.*  Advisers FGD

Not breastfeeding was seen by the breastfeeding advisers as a sign of not loving the child. The advisers alleged that they would chase out of the village any mother who refused to breastfeed.

*Participant 2: If you don’t want to breastfeed go far away but not in our village, because we Kaumas we love our children.* Advisers’ FGD

*Participant 4: What of the expressed milk*

*Participant 1: It smells*

*Participant 4: It is because it has not reached here that’s why you are saying it is hard*

*Participant 5: No, they don’t like the child.* Advisers FGD

The mothers also felt it was better to breastfeed on demand and return home from work or school rather than deny the child opportunities to feed from the breast.

*Participant 3: You are also not allowed to give him cow’s milk; in the morning before you leave you express enough for the day or if you work near to where you stay, enough to feed him until lunch time. Put it in a plastic feeding bottle and he uses your milk until he is 6 months or the baby…*

*Participant 2: Or you try your best to run home to breastfeed your baby when you break, there is no need of expressing*

*Participant 3: I want to say it’s hard for a young baby who has to feed like 6, 7, 8 times in a day. So, if you’ll be doing that you’ll be denying his rights. You breastfeed him when you are there and any other time he wants to feed.* Mothers FGD

### Lack of knowledge about expressing, storing and giving expressed breastmilk

Some of the mothers compared the process of expressing milk to milking a cow and did not wish to try it.

*Participant 5: The mother is there and he doesn’t want to suck, that’s why the milk is expressed and he is given using a spoon.*

*Participant 3: You express as if you are a cow, I cannot do that.* Mothers FGD

There were concerns on how to store the milk but also the nature of milk was raised as a potential objection by the older women, with breastmilk considered as coming from blood or even being part of the person.

*Participant 5: Where will she put it?*

*Participant 1: And it is blood, it circulates, if you wash with hot water it flows*

*Participant 4: If it flows is when she will express then where will she keep the milk? The milk will remain good for the baby to drink the whole day?*

*Participant 2: You mean the breast I express like this…then I keep?*

*Participant 4: To keep in the thermos or where do I keep it?*

*Participant 3: In the cup*

*Participant 2: You boil it?*

*Participant 1: And can it be boiled?*

*Participant 3: You will be cooking a person now* Advisers FGD

Most of the groups had never seen a baby being given expressed breast milk. One mother however did report that she had seen expressed breastmilk given to a sick baby who was admitted to the nearby health centre.

*Participant 2: It isn’t sensible.*

*Participant 1: I’ve never heard nor seen it, what I know is to buy cow’s milk, boil it and give it to the baby but people’s milk?* Mothers FGD

A common theme was the need to express a large enough volume to give to the baby throughout the day.

*Participant 2: Where will it be expressed, in a cup or?*

*Participant 1: I haven’t heard.*

*Participant 2: Until you get a thermos full?*

*Participant 3: Then you need to have enough milk.*

*Participant 2: Even if you have enough milk…*

*Participant 1: I haven’t heard of someone’s milk being expressed.*

*Participant 2: Like me, he gets satisfied by using one breast but you cannot express to satisfy him for the whole day.* Mothers FGD

### Negative attitudes towards expressed breastmilk

There was some reluctance from the older women to handle another person’s milk.

*Participant 5: We see it is wrong*

*Participant 1: Someone’s blood, I am surprised*

*Participant 3: I don’t want to touch it*

*Moderator: You don’t want to touch it*

*Participant 5: I don’t want to touch yes* Advisers FGD

### Traditional customs around expressing breastmilk

Most group members did not have any experience of giving expressed breastmilk to infants. They described practices of expressing and discarding milk when the mother or baby was ill, to relieve discomfort from engorgement or after the baby had died.

*Participant 3: Maybe seeing one is expressing it when she has stopped him from breastfeeding and maybe the breasts are full, then you can express it and throw it away.*

*Participant 6: Or sometimes you wake up and the breast is full and the milk is watery, that’s when you are allowed to express it in a container then you throw it away, but putting it in a container to give it to the baby, ah! That’s news.* Mothers FGD

*Participant 4: Maybe he has mumps he can’t suck.*

*Participant 1: If the baby is not around (alive) that is when you can express, but if he is around you can’t*. Advisers’ FGD

The tradition of expressing into a coconut shell for disposal came up frequently in the discussions but most participants were not sure why there had to be ashes or a hole in the bottom of the shell.

*Participant 3: The milk will be flowing, there is the thick milk, and the watery milk which is not good*

*Moderator: What do you do with the watery milk?*

*Participant 3: Traditionally, you express the milk into a coconut shell with a hole and some ashes in it, then you throw it in the rubbish pit and you are not allowed to look behind after throwing it*

*Moderator: Why a coconut shell with a hole on it?*

*Participant 3: I don’t know, I didn’t ask.* Mothers FGD

If the expressed milk was contaminated by a fly landing on it, the participants feared that the baby would be adversely affected even though it wasn’t given to the child.

*“Participant 4: The one who told me to express using a coconut shell with a hole at the bottom, she did not tell me the reason behind it; she only said that the milk should not be drunk by insects, but how will I throw it so that the milk is not drunk by insects?”* Advisers’ FGD

*“Participant 3: I heard that if you don’t express the milk in a coconut shell with some ashes in, it will cause diarrhoea to the baby.”* Mothers FGD

## Discussion

In the group discussions breastfeeding was considered the best way to feed young babies but alternative fluids were frequently given before the recommended age of 6 months. Caretakers, often grandmothers, were likely to give cows’ milk or maize porridge if the mother was away for more than a few hours and the baby started crying. There was little knowledge about or experience of expressing, storing and giving expressed breastmilk and there was resistance, especially from older women, to the concept of storing it and giving to the baby. Traditional customs on disposal seem to suggest a belief that milk must be carefully handled to avoid contamination by insects even if it is discarded or else the baby might suffer harm.

The strengths of this research are that it was carried out by research assistants and CHWs from the same area as the participants so that they could encourage free discussion about local health beliefs and practices around the time of birth and early infancy. The local researchers assisted the principal and co-investigators to understand the historical and cultural context of infant feeding practices. Discussion groups consisted of the mothers’ most trusted advisers who were often also the infants’ caretakers, thus indicating potential obstacles to giving expressed breastmilk.

The limitations of the study include the possibility of bias in composition of the mothers FGDs. The lag time between 2 and 20 months from answering of questionnaires to FGDs meant that some mothers had married and moved away, and others with paid employment or who had returned to school or college could not participate.

Although the rural population of Jaribuni has similar problems of poverty, low education level and lack of paid jobs as other coastal areas of Kenya, some of the findings about attitudes to handling of expressed milk may not be generalizable outside the county. Nevertheless, the high prevalence of teenage mothers in Kenya, government policy on mothers ‘right to continued education and maternity leave for only 3 months for those in paid employment make research, into enabling infants to receive their mothers’ milk whilst separated from them, a priority area for improving nutritional adequacy of infants ‘diets.

### Schoolgirl mothers

Our main study sample included a large proportion of adolescent mothers who had dropped out of formal education due to an unplanned pregnancy. Girls who become pregnant whilst at school are supposed to return to complete their studies after their babies are born and the children looked after at home “to avoid interruption of the girls’ studies.” [9]. The age of the baby at the time of the mother’s return to formal education is not clear from the Kenyan Government School Health Policy (“At the appropriate time the adolescent mothers may seek readmission to the same school or if they so wish join other schools” page 23) but this is interpreted locally by school head teachers and sub-county education coordinators. A further consideration is that family members wish a girl to return during the same school year so as not to forfeit school fees that have already been paid.

Some girls might have to walk for an hour each way between home and school meaning that they are separated from their babies for 10 hours or more on school days. Very few rural families living in the tropical climate of Kilifi possess fridges and the storage of expressed breastmilk in ambient temperatures (up to 25 °C) is not recommended for more than 6–8 hours [10]. One potential way of increasing storage time of expressed milk in homes without a fridge may be to heat treat it. This has been tested in the context of areas with high HIV transmission rates. Flash-heating to pasteurize expressed breastmilk to kill HIV and other pathogens has been evaluated in South Africa and has been shown not to adversely affect the bacteriostatic properties of the milk [11]. Human milk was heated to a typical peak temperature of 72.9 °C by placing it in a glass peanut butter jar in an aluminium pan of water brought almost to the boil [11]. In Kilifi HIV prevalence in women is 6.4% (much lower than in South Africa), so the main aim is to improve the keeping qualities of the milk [12]. More research into the method using local readily available containers is required to test the potential benefit of pasteurizing milk for babies of working mothers in Kilifi.

Research on home heat treated breastmilk in Tanzania showed that some mothers did not continue the procedure for long as it was considered too much work and it was easier to give alternative fluids [13]. The same barriers might be encountered in Kilifi when cooking is done on firewood or charcoal and cow’s milk or maize porridge is easily available to the household. Lack of knowledge of the superiority of human milk over other sources of nutrition may play a role locally. There should be more emphasis on educating mothers and caretakers on the immunological and nutritional benefits of giving human milk.

### Working mothers

In rural areas mothers may work on family farms or be self-employed, e.g. as traders in local markets, sometimes necessitating the child to be left with a caretaker. In poor families, this would be the child’s grandmother or an adolescent female relative. In better off families, a nanny might be employed.

In 2017 the Health Act no. 21 was passed, requiring all employers to establish breastfeeding stations with the necessary equipment and facilities for working mothers to breastfeed and express breastmilk for their infants [14]. Employers must provide additional break intervals for nursing mothers in addition to the regular times off for meals to breastfeed or express milk. The breastfeeding period must not exceed 1 hour for every eight working hours.

### Lactation support as part of routine health services

Breastfeeding counsellors are a scarce resource in Kenya though there are several countries in Sub-Saharan Africa where trained lay peer supporters have been used to give individual breastfeeding counselling and to establish breastfeeding support groups for mothers in the community [15]. There is a need to integrate information about giving expressed breastmilk into exclusive breastfeeding promotion and counseling into routine care at antenatal, postnatal and child welfare clinics, as well as during home visits by community health workers and breastfeeding peer supporters [16].

## Conclusions

Expressing breastmilk for feeding infants when the mother and baby are separated is a new concept in this population of rural mothers. More promotion and practical skills training in hand expression, safe breastmilk storage and feeding of expressed breastmilk by caretakers would help mothers to maintain their milk supply whilst continuing their education and work activities.

## Abbreviations

CHW: Community health worker
FGD: Focus group discussion
KEMRI: Kenya medical research institute
UNICEF: United Nations children’s fund
WHO: World Health Organization
